# Hypocapnia delays subsequent bupivacaine cardiotoxicity in rats under sevoflurane anesthesia

**DOI:** 10.1186/2193-1801-3-371

**Published:** 2014-07-21

**Authors:** Shuchun Yu, Toshiaki Mochizuki, Takasumi Katoh, Hiroshi Makino, Yuya Kawashima, Soichiro Mimuro, Shigehito Sato

**Affiliations:** Department of Anesthesiology and Intensive Care, Hamamatsu University School of Medicine, 1-20-1, Handa-yama, Higashi-ku, Hamamatsu-City, 4313192 Japan; Department of Emergency and Disaster Medicine, Hamamatsu University School of Medicine, 1-20-1, Handa-yama, Higashi-ku, Hamamatsu-City, 4313192 Japan

## Abstract

**Background:**

Hypocapnia induced following the accidental intravenous infusion of a local anesthetic can mitigate anesthetic toxicity, but the effects of hypocapnia induced prior to local anesthetic infusion are unknown. In this study, we examined the effects of prior hypocapnia on bupivacaine-induced cardiotoxicity in rats.

**Methods:**

Eighteen Sprague–Dawley rats were randomly divided into two groups: one receiving sevoflurane with normal ventilation (Control Group) and the other receiving sevoflurane with hyperventilation to induce hypocapnia (Hypocapnia Group). After 30 min, both groups received continuous intravenous infusions of 0.25% bupivacaine at 2 mg · kg^−1^ · min^−1^. The time taken to reach 25% and 50% reductions in heart rate (HR; HR-25%, HR-50%) and mean arterial pressure (MAP; MAP-25%, MAP-50%) from the start of bupivacaine infusion were recorded. The difference between HR-25% and MAP-25% was calculated. The times of the first ventricular premature beat (VPB) and final systole were also recorded.

**Results:**

In the Hypocapnia Group, HR-50%, MAP-25%, and MAP-50% were prolonged compared with the Control Group (*P* < 0.001). Furthermore, the interval between HR-25% and MAP-25% and the times between the first VPB and final systole were prolonged in the Hypocapnia Group (*P* < 0.001).

**Conclusion:**

In rats under sevoflurane anesthesia, prior hypocapnia delayed the onset of bupivacaine-induced cardiotoxicity. Prior hypocapnia should be avoided during continuous bupivacaine nerve block under general anesthesia, because it may delay the detection of cardiotoxicity.

## Introduction

Early signs and symptoms of local anesthetic toxicity include tinnitus, visual and auditory disturbances, restlessness, slurred speech, and muscle tremors (Strichartz and Berde 2005). However, these symptoms are often masked during general anesthesia and are typically noticed only after the signs of cardiotoxicity occur, such as ventricular premature beat (VPB) and/or hypotension. A recent review of the literature studies on of local anesthetic toxicity published over the past 30 years concluded that decreased heart rate (HR) and mean arterial pressure (MAP) may be represent important early signs of local anesthetic toxicity, potentially occurring in half of all cases (Di Gregorio et al. 2010).

Migration of the epidural catheter from the epidural space into a blood vessel, e.g., an epidural vein, is rare, but causes serious complications during continuous epidural analgesia. Several large observational studies reported incidences of migration of the epidural catheter into a blood vessel of 0–0.67% and 0.25% (Wu and Ouanes 2013; Pan et al. 2004). Accidental intravenous local anesthetic infusion due to catheter migration can cause sudden cardiovascular collapse (Wong et al. 2010; Khan and Khadijah 2008). In such cases, hyperventilation to induce hypocapnic alkalosis was proposed as a useful maneuver to combat local anesthetic toxicity (Porter et al. 2000. Mechanical ventilation with high tidal volumes (10 mL · kg^−1^–15 mL · kg^−1^ predicted body weight) has historically been recommended to prevent hypoxemia and atelectasis during general anesthesia (Bendixen et al. 1963; Kilpatrick and Slinger 2010); hence, some patients may already be hypocapnic prior to accidental local anesthetic exposure. However, it is unknown whether prior hypocapnia influences local anesthetic intoxication. In this study, we examined whether prior hypocapnia during general anesthesia delayed the onset of subsequent bupivacaine-induced cardiotoxicity.

## Methods

The experimental protocol was approved by the Institutional Animal Care and Use Committee of the Hamamatsu University School of Medicine (Hamamatsu, Japan; Permit No. 2009033). Eighteen male Sprague–Dawley rats, weighing 370 g–425 g, were randomly divided into two groups (*n* = 9 per group): those receiving sevoflurane alone (Control Group) and those receiving sevoflurane with hypocapnia (Hypocapnia Group). The trachea was cannulated by tracheotomy following the induction of sevoflurane anesthesia. Rats were placed on a ventilator (Model 683; Harvard Bioscience Inc., Holliston, MA, USA), and maintained at 50 breaths · min^−1^ and a fraction of inspired oxygen of 1.0. Polyethylene catheters (SP31; Natsume Co., Ltd., Tokyo, Japan) were placed in the right femoral artery and vein to monitor the MAP and for drug infusion, respectively. The left external jugular vein was cannulated to monitor central venous pressure (CVP) and for drug infusion. The effective dose for 50 percent of the group of sevoflurane in rats is 2.8%, which corresponds to the minimum alveolar anesthetic concentration determined using the tail-clamp technique (Taheri et al. 1991). Therefore, anesthesia was maintained with 2.8% inspired sevoflurane for the entire experimental period.

Rats were allowed to stabilize for 20 min following surgical preparation. The tidal volume was set at 6 mL · kg^−1^–8 mL · kg^−1^ in the Control Group and 11 mL · kg^−1^–12 mL · kg^−1^ in the Hypocapnia Group. Electrocardiograms, HR, MAP, and CVP were continuously recorded (PowerLab; ADInstruments, Bella Vista, NSW, Australia). Rectal temperature was maintained at 37°C–38°C throughout the experimental period using a heating pad. After 30 min of hyperventilation in the Hypocapnia Group or 30 min of ventilation in the Control Group, both groups were continuously infused with 0.25% bupivacaine at 2 mg · kg^−1^ · min^−1^ through the right femoral vein until the conclusion of the study. Prior to the injection of bupivacaine, arterial blood was sampled for blood gas analysis.

The time taken to reach 25% and 50% reductions in HR (HR-25%, HR-50%) and MAP (MAP-25%, MAP-50%) from the start of bupivacaine infusion were measured after the conclusion of the study. The difference between HR-25%, and MAP-25% was calculated to estimate the delay between the onset of HR decrease and myocardial contractile depression. The times of the first VPB and final systole were also measured after the conclusion of the study.

### Statistical analysis

All values are expressed as means (±standard deviation [SD]). The time required for HR and MAP to decline to 25% and 50% of their baseline values are expressed as means (±SD). Means and mean differences were compared by two-way between-subjects analysis of variance (ANOVA). When the between-subjects ANOVA was significant (*P* < 0.05), a *post-hoc* Tukey–Kramer test was applied using Aabel 3 software (Hulinks Inc., Tokyo, Japan).

## Results

There were no significant differences in body weight, initial HR, initial MAP, or initial CVP between the groups (Table 1). Only rats ventilated at a tidal volume of 11 mL · kg^−1^–12 mL · kg^−1^ (Hypocapnia Group) exhibited hypocapnia (partial pressure of carbon dioxide [pCO_2_] = 30 [±3] mm Hg; Table 2).

**Table 1:** **Baseline body weight and hemodynamic parameters**

	Control group (n =9)	Hypocapnia group (n = 9)
Weight (g)	393 (13)	393 (15)
HR (bpm)	354 (32)	326 (20)
MAP (mmHg)	106 (20)	97 (18)
CVP (mmHg)	3.7 (1.1)	4.0 (0.6)

HR, heart rate; MAP, mean arterial pressure; CVP, central venous pressure.

Data are presented as means (±standard deviation).

**Table 2:** **Blood gas analysis and hemodynamic variables before infusion of bupivacaine**

	Control group (n = 9)	Hypocapnia group (n = 9)
pH	7.42 (0.02)	7.53 (0.02)*
pO_2_ (mmHg)	449 (87)	425 (63)
pCO_2_ (mmHg)	43 (5)	30 (3)*
HR (bpm)	342 (29)	319 (18)
MAP (mmHg)	110 (18)	108 (15)
CVP (mmHg)	4.3 (1.0)	4.4 (0.8)

HR, heart rate; MAP, mean arterial pressure; CVP, central venous pressure.

Data are presented as means (±standard deviation).

*P < 0.001 versus Control Group.

The time from the onset of bupivacaine infusion to HR-25% was not prolonged in the Hypocapnia Group compared with the Control Group (305 [±137] s versus 298 [±171] s; *P* > 0.5). However, the time from onset of bupivacaine infusion to HR-50% was prolonged in the Hypocapnia Group compared with the Control Group (1818 [±347] s versus 818 [±357] s; *P* < 0.001; Table 3). The times required to reach MAP-25% and MAP-50% were prolonged in the Hypocapnia Group compared with the Control Group (MAP-25%: 1739 [±779] s versus 330 [±199] s; *P* < 0.001 and MAP-50%: 2319 [±440] s versus 1111 [±431] s; *P* < 0.001; Table 3).

**Table 3:** **The time and total doses of bupivacaine infused to reach HR-25%, HR-50%, MAP-25% and MAP-50% from the start of bupivacaine infusion**

		HR-25	HR-50	MAP-25	MAP-50
Control group (n = 9)	Time(s)	298 (171)	818 (357)	330 (199)	1111 (431)
	Total doses of bupivacaine infused (mg · kg^−1^)	9.9 (5.7)	27.3 (11.9)	11.0 (6.6)	37.1 (14.4)
Hypocapnia Group (n = 9)	Time(s)	305 (137)	1818 (347)*	1739 (779)*	2319 (440)*
	Total doses of bupivacaine infused (mg · kg^−1^)	10.1 (4.6)	60.6 (11.5)*	58.0 (26.0)*	77.3 (14.7)*

HR-25%, HR-50%, MAP-25%, and MAP-50% are defined as the time taken to reach 25% and 50% reductions in heart rate (HR) and mean arterial pressure (MAP) from the start of bupivacaine infusion, respectively. Total doses of bupivacaine infused to reach each time point are also described. HR-50%, MAP-25%, and MAP-50% were significantly prolonged in the Hypocapnia Group compared with the Control Group. Similarly, total doses of bupivacaine infused were higher in the Hypocapnia Group than in the Control Group at HR-50%, MAP-25%, and MAP-50%.

*P < 0.001 versus Control Group.

**Figure 1:**
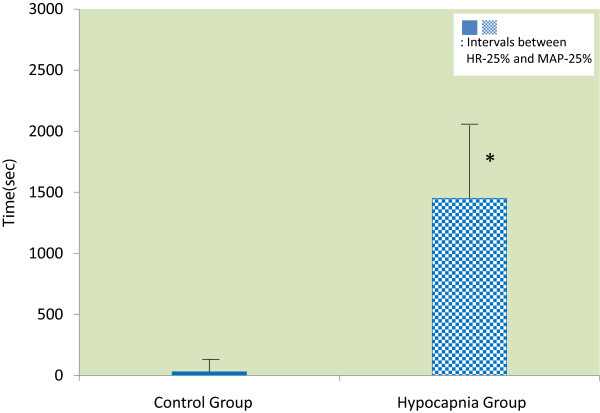
**The interval between HR-25% and MAP-25%.** The interval between HR-25%, and MAP-25% was significantly prolonged in the Hypocapnia Group compared with the Control Group. HR-25%, 25% reduction in heart rate; MAP-25%, 25% reduction in mean arterial pressure. *P < 0.001 versus Control Group.

**Figure 2:**
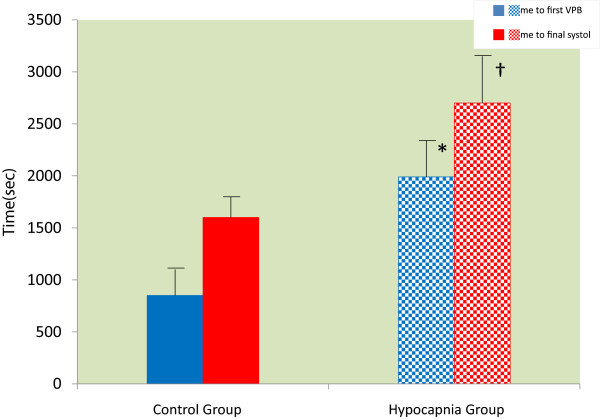
**Time to the first ventricular premature beat and to the final systole.** The times to the first ventricular premature beat (VPB) and final systole were prolonged in the Hypocapnia Group compared with the Control Group. *P < 0.001 versus time to the first VPB in the Control Group. †*P* < 0.001 versus time to the final systole in the Control Group.

The Hypocapnia Group also demonstrated a longer interval between HR-25% and MAP-25% compared with the Control Group (1434 [±780] s versus 32 [±120] s; *P* < 0.001; Figure 1). The times of the first VPB and final systole were also prolonged in the Hypocapnia Group compared with the Control Group (first VPB: 1938 [±318] s versus 848 [±310] s; *P* < 0.001 and time to final systole: 2675 [±314] s versus 1593 [±250] s; *P* < 0.001; Figure 2).

## Discussion

Decreases in HR and MAP are important early signs of local anesthetic toxicity in the myocardium (Di Gregorio et al. 2010; Scott et al. 1989; Mauch et al. 2011), which can progress to asystole or malignant ventricular arrhythmia (Di Gregorio et al. 2010). Thus, it is vital that any inadvertent intravenous infusion of local anesthetic, as can occur with catheter migration, be recognized as early as possible. Our results demonstrate that prior hypocapnia delayed these early signs of bupivacaine-induced cardiotoxicity in rats under sevoflurane anesthesia. Although this delay suggests the partial mitigation of cardiotoxicity due to hypocapnic alkalosis, any delay in bradycardia or hypotension permits an overall greater infusion of local anesthetic into the circulation, ultimately exacerbating cardiotoxicity. Thus, hypocapnia and associated alkalosis should be avoided during procedures requiring general anesthesia and local nerve blocks.

Hypocapnic alkalosis induced by hyperventilation may reduce the cardiotoxicity induced by systemic local anesthetics (Porter et al. 2000; Englesson and Grevsten 1974). Hypocapnia induced prior to intracoronary infusion of ropivacaine in anesthetized dogs decreased the magnitude of cardiotoxicity, implying that hypocapnic alkalosis may be a useful adjunct (Porter et al. 2000). In addition, hypocapnia reversed the local anesthetic-induced suppression of baroreflex sensitivity (Watanabe et al. 1997). In contrast, bupivacaine-induced cardiotoxicity was enhanced by prior acidosis and hypercapnia (Englesson and Grevsten 1974; Rosen et al. 1985). Increased blood pH converts the charged (channel-blocking) form of the local anesthetic to the uncharged form, which has a much lower channel affinity and diffuses into the cell more easily. Hypocapnia increases intracellular pH, which decreases the intracellular concentration of the active, charged form of the local anesthetic (Strichartz and Berde 2005). This mechanism may also explain the delayed bupivacaine-induced cardiotoxicity following prior induction of hypocapnia.

The interval between HR-25% and MAP-25% was markedly prolonged (44-fold) by prior hypocapnia. We assume that this interval is equivalent to the time from the onset of primary toxic symptoms until the start of myocardial contractile depression. This delay permits more bupivacaine to enter the circulation (in the scenario of accidental infusion), making it more difficult to treat patients with hypocapnia than those with normal blood pCO_2_ and pH. Historically, mechanical ventilation with high tidal volumes, i.e., >10 mL · kg^−1^, has been recommended to prevent hypoxemia and atelectasis in anesthetized patients (Bendixen et al. 1963). In contrast, in current practice, lung protective strategies, including mechanical ventilation with lower tidal volumes than historic ventilation criteria, are recommended to minimize barotrauma in the lung, which can cause acute respiratory distress syndrome (Kilpatrick and Slinger 2010). Hyperventilation by mechanical ventilation with high tidal volumes may enhance the risk of local anesthetic toxicity. In the Hypocapnia Group, the time to VPB was extended by 68%, and the time to asystole was extended by 128%, compared with the Control Group. These relative delays were considerably shorter than those between 25%-HR and 25%-MAP, possibly because the protocol was such that the amount of local anesthetic administered was already in the toxic range by the time MAP-25% was attained. Hyperventilation by mechanical ventilation with high tidal volumes should not be recommended in cases where barotrauma of the lung must be minimized, or when excessive infusion of local anesthetics into the circulation has occurred following catheter migration into a blood vessel.

We showed that hypocapnia under sevoflurane anesthesia may be dangerous during the continuous infusion of local anesthetics, because it delays the recognition of inadvertent toxicity due to catheter migration. However, this investigation is limited by the fact that all the experiments were conducted under hyperoxic conditions. Future studies must examine these effects under a range of oxygenation conditions. Furthermore, we did not measure blood concentrations of bupivacaine in this study. Because HR-25%, HR-50%, MAP-25%, MAP-50%, time of the first VPB, and time of the final systole were calculated after the conclusion of the study, it was impossible to obtain blood samples at these time points.

Concerning the time taken for recovery from HR-50% to the baseline HR after bupivacaine cessation, we expect that the recovery time be prolonged in the Hypocapnia Group compared with the Control Group, according to our previous study (Mochizuki and Sato 2008). Similar results are anticipated for the time required for recovery from MAP-25% or MAP-50% to the baseline MAP. Further study is necessary in this regard.

Aging degenerates the cardiac conduction systems, accelerating the occurrence of ion channel dysfunction, conduction delay, and arrhythmia (Park and Fishman 2011). In many studies on the systemic toxicity of local anesthetics, the weight of the Sprague–Dawley rats used ranged from approximately 300 g to 350 g. In this study, the weight of the Sprague–Dawley rats used ranged from 370 g to 425 g. This means that the rats used in this study were older than those weighing 300 g–350 g used in similar studies. Therefore, the absolute values of HR-25%, HR-50%, MAP-25%, MAP-50%, time of the first VPB, and time of the final systole in this study may have been influenced by aging compared with the results of studies using younger rats. However, because the effects of hypocapnia on local anesthetics are independent of aging, we believe that the results of this study are relevant to the use of local anesthetics in clinical practice.

In conclusion, hypocapnia during general anesthesia with sevoflurane delayed the onset of bupivacaine-induced cardiovascular toxicity in rats. Despite this protective effect, hypocapnia is not recommended during continuous nerve blocking using local anesthetic under general anesthesia, because it may facilitate a greater infusion of toxins and, ultimately, cause greater cardiovascular injury.
